# A sugary addition to the urea cycle

**DOI:** 10.1093/jmcb/mjac027

**Published:** 2022-04-23

**Authors:** Chad Slawson

**Affiliations:** Department of Biochemistry and Molecular Biology, University of Kansas Medical Center, Kansas City, KS 66160, USA

In a recent publication, Wu et al. (2022) asked a simple premise: how does aging impact *O*-GlcNAcylation in the liver? What they discovered, however, requires us to ask a more fundamental question: how does aging alter nutrient-sensing pathways and the messages they communicate? In order to answer this question, we must remember that nutrient sensing is hinged on post-translational modifications (PTMs) and the availability of nutrients necessary for producing PTM metabolites. Adenosine monophosphate (AMP)-regulated protein kinase, which is sensitive to the ratio of AMP to adenosine triphosphate, and mammalian target of rapamycin, an anabolic kinase regulated by mitogen signaling and amino acid availability, are two of the most studied representations of this phenomenon for phosphorylation modifications (Cork et al., 2018). When we consider these kinds of signaling pathways as well as the ones directed by other common PTMs like acetylation or ubiquitinoylation, *O*-GlcNAcylation proves to be quite unique. *O*-GlcNAc is a single *N*-acetyl-glucosamine molecule attached to cytoplasmic, nuclear, or mitochondrial proteins by the actions of a single enzyme, *O*-GlcNAc transferase (OGT), and removed by a single enzyme, *O*-GlcNAcase (OGA). Like any abundant PTM, *O*-GlcNAc regulates a myriad of cellular functions and behaviors. Importantly, OGT uses uridine diphosphate (UDP)-GlcNAc synthesized in the hexosamine biosynthetic pathway as the metabolic substrate for the glycosyltransferase reaction (Cork et al., 2018). As such, UDP-GlcNAc concentrations are dependent on glucose, amino acid, fatty acid, and nucleotide levels, making *O*-GlcNAcylation levels a sensitive gauge of the availability of these macromolecules for the cell (Cork et al., 2018). Thus, changes in nutrient levels will impact OGT function and quickly lead to adaptive changes in most to all cellular processes. Returning to the work by Wu et al. (2022), let us once again ask a simple question: how does aging impact *O*-GlcNAcylation? As it turns out, *O*-GlcNAc impacts aging through its regulation of the urea cycle, a critical aspect of cellular metabolism.

So, what exactly does Wu et al. (2022) find? Using young (4 months) and aged mice (24 months), the researchers observe a global increase in *O*-GlcNAcylation in several tissue types. Using *O*-GlcNAc affinity enrichment, they identify a substantial number of liver proteins that demonstrate a change in *O*-GlcNAcylation status based on age. Leading the list of aging-enriched glycoproteins in the liver were proteins associated with classic hallmark of aging pathways (Lopez-Otin et al., 2013) such as mitochondrial dysfunction, loss of proteostasis, and deregulated nutrient sensing. Interestingly, carbamoyl phosphate synthetase 1 (CPS1), the enzyme responsible for converting ammonia to carbamoyl phosphate in the first step of the urea cycle, becomes increasingly *O*-GlcNAcylated in the livers of the older mice. Turning to cell line models, the researchers then determine that CPS1 is a substrate for both OGA and OGT. Next, they modulate nutrient levels to maintain increased global *O*-GlcNAcylation by increasing the glucose flux or inhibiting OGA; in both methods, they find high *O*-GlcNAc levels occurring in concurrence with decreased CPS1 activity.

To explore the possibility of *O*-GlcNAc as a direct modifier of CPS1 activity, the researchers perform mutagenesis experiments to investigate three *O*-GlcNAcylation sites mapped on CPS1; the final outcome suggests that Ser537 is the key *O*-GlcNAcylation site on CPS1, the Ser537Ala mutant resulting in higher activity than the wild-type counterpart. Finally, this study shows that mice with loss of liver OGT have a robust ability to detoxify ammonia under fasting conditions. Overall, these data strongly show that *O*-GlcNAcylation is a critical regulator of CPS1 function and, in turn, the urea cycle.

CPS1 is the rate-limiting enzyme of the urea cycle and catalyzes three reactions to convert ammonia into carbamoyl phosphate (Nitzahn and Lipshutz, 2020). Since ammonia is highly toxic to ureotelic animals, CPS1 mutations leading to loss of function are rare and lethal, although mutations that lead to late onset urea cycle disorders can be treated therapeutically. Urea cycle deficiencies are linked to numerous diseases and contribute to higher ammonia levels in obese patients (Nitzahn and Lipshutz, 2020). Intriguingly, Wu et al. (2022) link loss of urea cycle function to nutrient excess through *O*-GlcNAcylation.

With all of this said, *O*-GlcNAc is the devil. At least, it can be a not-so-sweet subject to study for several reasons. Since *O*-GlcNAc is highly abundant, OGT and OGA are ubiquitously expressed, and the modification is involved in regulating every major cellular function, teasing out mechanistic information from animal studies can be extremely difficult. Although the OGT liver knockout studies are elegant, it is hard to know for sure whether the loss of OGT improves ammonia clearance after fasting specifically because of increased CPS1 activity. OGT loss could be influencing numerous pathways or potentially other regulatory PTMs on CPS1 such as succcinylation (Nakagawa et al., 2009; Du et al., 2011). Yet, OGT knockout methods in tissue or cell lines are the most powerful technique in an *O*-GlcNAc researcher's toolbox to ascertain mechanistic information, i.e. until clustered regularly interspaced short palindromic repeats (CRISPR) technology advances enough for rapid and cost-effective generation of site-specific mutations of *O*-GlcNAc sites in endogenous tissue. Unfortunately, OGT has a highly promiscuous active site that might simply modify a serine or threonine near the mutated *O*-GlcNAc site on CPS1, a phenomenon which has been observed in FOXO studies (Fardini et al., 2015).

Still, Wu et al. (2022) manage to generate some chewy but intriguing food for thought as we consider the old adage of ‘which came first, the chicken or the egg?’ Do disruptions in *O*-GlcNAc nutrient-sensing drive aging or does aging drive *O*-GlcNAc disruption? Likely, the yolk of the answer is a mix of these two possibilities. Most assuredly, impaired *O*-GlcNAc nutrient sensing will have a detrimental effect on organismal lifespan, potentially by disturbing critical metabolic pathways like the urea cycle. What is the long-term impact of increased CPS1 *O*-GlcNAcylation on urea cycle function and aging? Can reduced CPS1 *O*-GlcNAcylation slow aging and improve ammonia-handling? Could increased *O*-GlcNAcylation of CPS1 silently promote disease phenotypes? A simple question by Wu et al. (2022) leads to these new and exciting questions about nutrient sensing and aging, but we have to ask many more questions if we want to uncover what *O*-GlcNAc nutrient sensing means in the grand scheme of aging. Here is to asking those questions as quickly as possible; after all, none of us are getting any younger.
